# Aménorrhée chimio induite chez une population marocaine: à propos d'une cohorte retrospective

**DOI:** 10.11604/pamj.2016.24.58.8892

**Published:** 2016-05-13

**Authors:** Sami Aziz Brahmi, Fatima Zahra Ziani, Seddik Youssef, Said Afqir

**Affiliations:** 1 Service d'Oncologie Médicale, Centre Hospitalier Mohammed VI, Oujda, Maroc; 2 Service d'Oncologie Médicale, Centre Hospitalier Hassan II, Fès, Maroc

**Keywords:** Breast cancer, chemotherapy, amenorrhea, Breast cancer, chemotherapy, amenorrhea

## Abstract

**Introduction:**

Le cancer du sein est un des cancers les plus fréquents chez la femme en pré ménopause et son traitement peut compromettre la fertilité. En effet, la chimiothérapie utilisée dans le cancer du sein peut conduire à une aménorrhée transitoire ou permanente chez les femmes non ménopausées.

**Méthodes:**

Nous avons mené une étude rétrospective au service d'oncologie médicale du CHU Mohammed VI d'Oujda sur une période de 3 ans allant de janvier 2009 à décembre 2011 incluant les patientes jeunes ayant un cancer du sein localisé, pour étudier l'incidence de l'aménorrhée chimio-induite (ACI), ainsi que les facteurs prédictifs intervenant dans sa survenue.

**Résultats:**

Dans notre série 74% de nos patientes ont présenté une ACI et 33.6% de nos patientes ont présenté une aménorrhée chimio-induite définitive. Plusieurs facteurs ont été étudiés à la recherche d’élément prédictifs de la survenue de l'aménorrhée. Concernant le facteur âge, l'analyse a montré que les femmes avec un âge supérieur à 40 ans étaient plus susceptibles de présenter une aménorrhée que celles avec un âge inférieur à 40 avec un pourcentage de 95,7% versus 56,1% avec une différence significative (p = 0.003).

**Conclusion:**

L'incidence dans notre étude de l'ACI semble comparable à celle retrouvé dans la littérature, l’âge dans notre étude est le facteur prédictif le plus prédominant dans sa survenue.

## Abstract (en)

**Introduction:**

Breast cancer is one of the most common cancers in premenopausal women and its treatment may affect their fertility. Indeed, chemotherapy used in breast cancer may cause transient or permanent amenorrhea in premenopausal women.

**Method:**

We conducted a retrospective study of young patients with localized breast canceri in the Department of Medical Oncology, Mohammed VI Inuversity Hospital, Oujda, Morocco over a 3-year period from January 2009 to December 2011. The aim of our study was to analyse the impact of chemotherapy-induced amenorrhea (CIA) as well as predictive factors for its occurrence.

**Results:**

In our series, 74% of patients had CIA and 33.6% of patients had definitive chemotherapy-induced amenorrhea. Several factors have been studied in search of predictive factors for amenorrhea occurrence. With regard to the age factor, our analysis showed that women over 40 were more likely to have amenorrhea than those aged less than 40 years (95.7% versus 56.1%), with a statistically significant difference (p = 0.003).

**Conclusion:**

In our study the incidence of ICA seems comparable to that found in the literature, while age is the predominant predictor of its occurrence.

## Introduction

Le cancer du sein est certainement la pathologie maligne la plus fréquente de la femme en âge de procréer et son traitement peut compromettre la fertilité. En effet, la chimiothérapie utilisée dans le cancer du sein peut conduire à une aménorrhée transitoire ou permanente chez les femmes non ménopausées. Nous avons mené une étude rétrospective au service d'oncologie médicale du CHU Mohammed VI d'Oujda sur une période de 3 ans allant de janvier 2009 à décembre 2011 incluant les patientes jeunes ayant un cancer du sein localisé. Nous vous proposons à travers ce travail, l’étude de l'incidence de l'aménorrhée chimio-induite, ainsi que les facteurs prédictifs intervenant dans sa survenue.

## Méthodes

Il s′agit d′une étude cohorte de type rétrospective réalisée sur une période de 3 ans allant de Janvier 2009 à Décembre 2011, et portant sur 104 femmes âgées de 44 ans et moins, traitées pour cancer du sein localisé au service d'oncologie médicale du CHU Mohammed VI d'Oujda.

### Critères d'inclusion

Femmes âgées de moins de 45 ans; cancer du sein localisé; patientes ayant reçu une chimiothérapie; cycles menstruels réguliers au diagnostic

### Critères d'exclusion

Femmes avec âge supérieur ou égal à 45 ans; cancer du sein métastatique; patientes n'ayant pas reçu de chimiothérapie; femmes ménopausées avant le début de la chimiothérapie. Dans notre étude on a définit une aménorrhée chimio-induite (ACI) par l'absence de règles pendant une durée de 6 mois ou plus après le début de la chimiothérapie. Notre étude avait pour but d'estimer l'incidence de l'aménorrhée chimio-induite et d’étudier les facteurs intervenant dans sa survenue. Des fiches d'exploitation détaillées ont été remplies pour chaque patiente. Les patientes ont été convoquées pour collecter les données notamment celles de l'aménorrhée chimio-induite.

### Analyse statistique

Le logiciel IBM SPSS STATISTICS version 21 a été utilisé pour l'analyse des données (données descriptives, effectifs et tableaux croisés, étude uni-variée) avec le test de khi-deux pour l'analyse des facteurs prédictifs de l'aménorrhée chimio-induite.

## Résultats

### Données épidémiologiques

Durant une période de 3 ans, de janvier 2009 à décembre 2011, 948 femmes atteintes de cancer du sein ont été recensé au service d'oncologie médicale CHU Mohammed VI d'Oujda, parmi elles, 104 patientes ont été inclues dans l’étude. L’âge moyen de nos patientes était de 38 ans, avec des extrêmes de 24 et 44 ans. L′âge de la ménarche a été précisé chez la totalité des femmes, avec des extrêmes de 11 à 16 ans, ce qui représente une moyenne de 13 ans. 91% des patients avaient un carcinome canalaire infiltrant exprimant les récepteurs hormonaux dans 77,8% des cas et le récepteur HER2 (human epithelial receptor 2) dans 18% des cas.

### Données sur le traitement

La chimiothérapie a été administrée selon deux modalités néoadjuvante chez 6 patientes et adjuvante chez 98 patientes. Les drogues de chimiothérapie utilisées étaient les anthracyclines (Epirubicine, adriamycine), les antimétabolites (5- fluoro-uracyle), les alkylants (cyclophosphamide) et les taxanes (paclitaxel et docetaxel), des cures espacées de 3 semaines étaient administrées avec un nombre de cures variant de 4 à 8 cures. La chimiothérapie a été administrée selon deux modalités: traitement par anthracyclines et alkylants seuls (protocole AC60 et FEC100); traitement séquentiel à base d'anthracycles et alkylants suivi de cures à base de taxanes (docetaxel et paclitaxel). Le Tableau 1 résume les protocoles de chimiothérapie utilisés. L'hormonothérapie a été indiquée chez 81 patientes, il s'agissait d'une hormonothérapie par Tamoxifène à une dose de 20mg par jour en une seule prise pour une durée moyenne de 5 ans. La thérapie ciblée par le Trastuzumab était indiquée chez 19 patientes.

**Tableau 1:** Protocoles de chimiothérapie utilisés dans notre série

Protocoles	nombre	Pourcentage %
6 AC 60	56	53
4 AC 60	2	1.9
6 FEC 100	4	3.8
3 AC 60 3 DOC	23	22
4 AC 60 4 DOC	7	6
3FEC100 3 DOC	5	4
4AC60 4 PACLI	7	6
TOTAL	104	100

C:Cyclophosphamide, A: Adriamycine, DOC: Docetaxel, F:Fluorouracile, E: Epirubicine PACLI: Paclitaxel.1

### Aménorrhée chimio induite

Dans notre série 77 de nos patientes ont présenté une aménorrhée chimio induite ce qui représente 74% des cas, alors que 27 patientes n'ont pas présenté d'aménorrhée (26% des cas). Après étude statistique descriptive, on a noté un pourcentage cumulé de 60,3% d'ACI après la 3^ème^ Cure (Tableau 2). Dans notre série 26 de nos patientes ont présenté une aménorrhée chimio-induite définitive ce qui représente 33,7% des cas, alors que le retour des règles a été enregistré chez 51 (66,7%) malades avec un délai médian de 12 mois et des extrêmes entre 6 et 36 mois. A noter que chez les patientes avec un âge inférieur à 35 ans (au nombre de 15 dans notre série), seulement 3 d'entre elles ont présenté une ACI, et toutes les 3 ont récupéré des cycles réguliers après 9 mois pour une patiente et 12 mois pour 2 autres. Plusieurs facteurs ont été étudiés à la recherche d’élément prédictifs de la survenue de l'aménorrhée notamment: l’âge, le type de chimiothérapie. Concernant le facteur âge, l'analyse a montré que les femmes avec un âge supérieur à 40 ans étaient plus susceptibles de présenter une aménorrhée que celles avec un âge inférieur à 40 avec un pourcentage de 95,7% vs 56,1% et de façon significative (p = 0.003). Donc dans notre série, l’âge supérieur à 40 ans est un facteur prédictif de survenue de l'ACI. Concernant le facteur chimiothérapie, la dose cumulée constituerait un facteur prédictif dans notre étude mais de façon non significative. En effet, 62 des patientes qui ont reçu 6 cures de chimiothérapie ont présenté une ACI (81,7%) versus 11 cas des 14 patientes ayant reçu 8 cures (14,4%) et 4 patientes ayant reçu 4 cures de chimiothérapie (3,8% des cas). L'analyse statistique n'a pas montré de différence significative (p = 1,4). Le type de chimiothérapie et les modalités d'administration ne semblent pas intervenir dans la survenue de l'ACI dans notre étude. Ainsi, chez 46 cas des patientes qui ont reçu une chimiothérapie à base de traitement séquentiel, on a enregistré 30 cas d'ACI (39%) versus 58,9% pour les patientes ayant reçu une chimiothérapie à base d'anthracyclines et agents mais cette différence n’était pas significative (p = 0,145). L'incidence de retour des règles semble aussi être influencer par l’âge dans notre étude mais de façon non significative, ainsi chez les femmes < de 40 ans ayant reçu un protocole séquentiel AC-taxane, l'incidence est de 79% chez les femmes moins de 40 ans et versus 45% pour celles plus de 40 ans. L'administration du trastuzumab ou du tamoxifène n’était pas un facteur prédictif de l'ACI dans notre étude. D'autres facteurs ont été étudiés notamment l’âge de ménarche et l'indice de masse corporelle mais ils ne seraient pas des facteurs prédictifs de survenue de l'ACI dans notre étude.

**Tableau 2:** Timing de l'aménorrhée dans notre série

Installation de l'aménorrhée	Nombre	Pourcentage %
**C1**	6	7,7
**C2**	9	11,6
**C3**	34	44,1
**C4**	13	16,8
**C5**	5	6,4
**C6**	10	12 ,9
**TOTAL**	77	100

## Discussion

La chimiothérapie utilisée dans le cancer du sein conduit à une aménorrhée transitoire ou permanente chez les femmes non ménopausées et l’âge est le principal facteur qui influence la reprise de cycles menstruels [1, 2]. Sur des coupes histologiques de patientes exposées à des traitements de chimiothérapie, les lésions vont d'une diminution de la densité des follicules à leur disparition complète avec présence d'une fibrose ovarienne [3]. Le nombre de follicules primordiaux est diminué et le stroma altéré [4]. En ce qui concerne la définition de l'ACI, chacune des études menées ont précisé la durée de l'aménorrhée, de 3 jusqu’à 12 mois d'absence des règles dans les suites d'un traitement par chimiothérapie du cancer du sein. L'incidence de l'ACI varie en fonction de l’âge des patientes, le protocole de chimiothérapie utilisé, les doses cumulées de chimiothérapie et l'ajout ou non de l'hormonothérapie [4]. Dans notre série, l'incidence globale de l'ACI était de 74% avec comme seuil une aménorrhée de 6 mois après administration de la chimiothérapie. Dans la série d'Amir et al, l'incidence de retour des règles chez les patientes ayant reçu un protocole AC est de 66% [5]. Dans notre série l'incidence de retour des cycles chez les patientes qui ont reçu le protocole AC était de 66,7%. Dans la même série d'Amir et al, l'incidence de retour des règles chez les femmes < de 40 ans ayant reçu un protocole séquentiel AC-taxane est de 85%, chez les femmes > à 40 ans ce taux devient 50%. Dans notre série, après une étude multi variée, ce taux est de 79% chez les femmes < 40 ans et de 45% (>40 ans). Donc l’âge constitue un facteur prédictif de l'allongement de la période d'ACI et aussi du retour ou non des règles [6]. Dans notre série 26 de nos patientes ont présenté une aménorrhée chimio-induite définitive ce qui représente 33,7% des cas, alors que le retour des règles a été enregistré chez 51(66,7%) malades avec des une durée moyenne de 12 mois et des extrêmes entre 6 et 36 mois. Les 26 patientes qui ont présenté une ACI définitive ont toutes un âge > 40 ans ce qui rejoint les données de la littérature (Tableau 3). Ce sont les agents alkylants qui ont été le plus souvent incriminés dans les phénomènes de toxicité gonadique [7, 8]. Le cyclophosphamide est l'agent de cette classe le plus utilisé en cancérologie mammaire, retrouvé dans la plupart des protocoles actuellement proposés en oncologie mammaire. Il a été de ce fait le plus étudié et sa toxicité est bien évaluée. Goldhirsch et al [9] ont montré qu'une administration l'endoxan entraînait 10% d'ACI, ce taux augmentant respectivement à 33 et 61% après 6 et 12 cures. En ce qui concerne les taxanes, peu de publications ont été dédié pour l’étude de leurs toxicités ovariennes, et les résultats sont le plus souvent contradictoires [10].

**Tableau 3:** Reprise des règles avec les différents protocoles de chimiothérapie

EQUIPES	CHIMIOTHERAPIE	% de reprise de règles
Goldhirsch 1990	Alkylants	39% <40 ans 5% >40 ans
Bines 1996	Alkylants	66%
Nabholtz 2002	Alkylants	67%
Nabholtz 2002	Alkylants et taxanes	48%
Roche 2006	Alkylants	28%
Roche 2006	Alkylants et taxanes	32%
Fornier 2005	Alkylants et taxanes	85% < 40 ans
Oktay 2005	Alkylants et taxanes	50% >40 ans
Notre série	Alkylants	66,7%
	Alkylants et taxanes	79% < 40 ans
	Alkylants et taxanes	45% >40 ans

La dose totale des cytotoxiques utilisés au cours de la chimiothérapie est un facteur déterminant de la réversibilité de l'insuffisance ovarienne chimio-induite [10]. De nombreuses études ont permis de préciser l'effet des doses cumulées sur la sévérité de l'IOC et son caractère réversible ou définitif [11]. Ainsi, 1 à 3 cycles de chimiothérapie entraînent une ACI dans 49,4% des cas. Ce taux est significativement augmenté à 60% après 4 à 6 cycles [10]. La toxicité gonadique du traztuzumab est mal connue, notamment du fait qu'il est fréquemment associé à des thérapeutiques adjuvantes telles que des polychimiothérapies et du tamoxifène. Le Tamoxifene contribue parfois au maintien de l'aménorrhée chimio-induite par augmentation du taux d'oestradiolémie et rétrocontrôle négatif sur l'axe hypothalamohypophysaire [11]. Dans son étude, l'auteur étudie les différentes probabilités de recouvrir des cycles menstruels après chimiothérapie et hormonothérapie par Tamoxifene. Après ajustement des variables utilisées: âge, poids, gravité, parité, âge des premières règles, tabac, alcool, hormonothérapie par Tamoxifene, type de chimiothérapie, emploi de trastuzumab, l'auteur conclut au fait que la persistance de l'aménorrhée est associée de façon significative à l’âge de la patiente et à la prise de Tamoxifene [11]. Une étude américaine présentée lors du congrès de la société américaine d'oncologie clinique (ASCO 2014) rapporte des résultats intéressants avec un agoniste de la GnRH, la goséréline pour préserver la fertilité des patients en cours de chimiothérapie. Au total, 257 femmes pré-ménopausées atteinte d'un cancer non hormonodépendant ont reçu un traitement par chimiothérapie contenant de la cyclophosphamide seule ou cette même chimiothérapie associée à la goséréline. Deux ans après le début de la chimiothérapie, 8% des femmes ayant reçu de la goséréline ont eu une ACI versus 22% pour les autres [12].

## Conclusion

Le cancer du sein est une maladie grave, son incidence chez les patientes jeunes marocaines est en augmentation. Le traitement de ce cancer dans sa forme localisée repose souvent sur une chimiothérapie qui présente avec un risque de diminution de la fertilité de ces patientes, essentiellement par altération de leur fonction ovarienne. L'aménorrhée chimio-induite dépend de plusieurs facteurs dont l’âge des patientes qui constitue le facteur le plus prédictif de son installation, le protocole de chimiothérapie utilisé et les doses cumulées de celle-ci peuvent influencer aussi l'installation et la durée de l'ACI. Cette aménorrhée peut être transitoire comme elle peut être définitive et par conséquent elle peut menacer la fertilité des femmes jeunes désirant une grossesse ultérieure.

### Etat des connaissances actuelle sur le sujet

- La chimiothérapie utilisée dans le cancer du sein présent un risque d'altération de la fertilité.

### Contribution de notre étude à la connaissance

- La première étude au niveau au Maroc qui traite de l'aménorrhée chimio induite, évalue son incidence et les facteurs de risque.
